# Supplementary material for: Restoration of visual function by transplantation of optogenetically engineered photoreceptors
Source: Nat Commun. 2019 Oct 4;10:4524. doi: 10.1038/s41467-019-12330-2 (PMC6778196; doi:10.1038/s41467-019-12330-2)
Supplement: Supplementary file 1 — Supplementary Information [file 41467_2019_12330_MOESM1_ESM.pdf]

Restoration of visual function by transplantation of optogenetically engineered photoreceptors

Garita-Hernandez et al.

Supplementary Figures

Supplementary Figure 1. NpHR expression in rod photoreceptors of donor mice.

Supplementary Figure 2. Transplanted NpHR-rod precursors are located in close apposition to the host INL and express the synaptic marker synaptophysin.

Supplementary Figure 3. Quantification of NpHR-expressing cells after transplantation of NpHR-rod precursors.

Supplementary Figure 4. NphR-expressing photoreceptor precursors transplanted into rd1 mouse respond to light.

Supplementary Figure 5. Halorhodopsin-triggered RGC responses in rd1 mice.

Supplementary Figure 6. Growth of the neuroepithelium in retinal organoids treated with FGF2 and the effect of Notch inhibition on retinal organoidogenesis and photoreceptor commitment.

Supplementary Figure 7. Physiological analysis of monolayer cultures derived from dissociated retinal organoids.

Supplementary Figure 8. Expression of human and photoreceptor markers in transplanted GFP^+ cells.

Supplementary Figure 9. Signal transduction from photoreceptors to the second order neurons in rd1 mouse transplanted with Jaws-photoreceptors.

Supplementary Figure 10. Jaws-triggered RGC responses in rd1 mice.

Supplementary Figure 11. The level of functional improvement is independent of the mouse host age at the time of transplantation.

Supplementary tables

Supplementary Table 1. A list of all mice used to generate figures, with specified strain, experimental group, experiment type, and ages at the time of transplantation and at the time of experiment

Supplementary Table 2. Media formulation.

Supplementary Table 3. List of TaqMan® Gene Expression ID Assays used for qRT-PCR

Supplementary Table 4. List of primary antibodies used for immunostaining

Supplementary figures

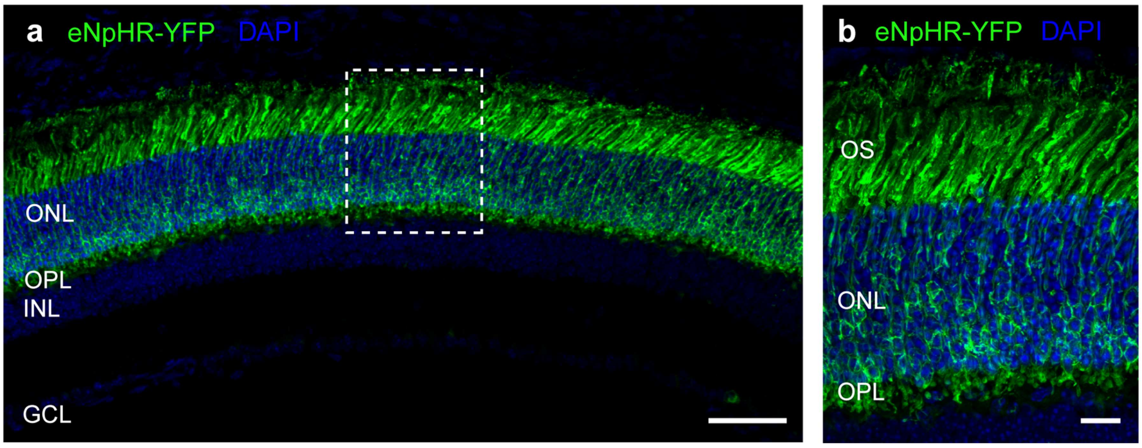

Supplementary Figure 1. NpHR expression in rod photoreceptors of donor mice. **a, b** A vertical section of a wild type mouse retina at P10, after AAV9-2YF-hRho-NpHR-YFP has been intravitreously injected at P2. As the mouse retina is vastly dominated by rod photoreceptors, we selected a promoter that drives gene expression in rods, in order to generate a high number of donor cells for transplantation studies (see also Busskamp et al., 2010 [1]). NpHR-expressing cells are shown in green, the sample was counterstained with DAPI. Scale bars are 50 μm (a) and 10 μm (b). OS – outer segments, ONL – outer nuclear layer, OPL – outer plexiform layer, INL – inner nuclear layer, GCL – ganglion cell layer.

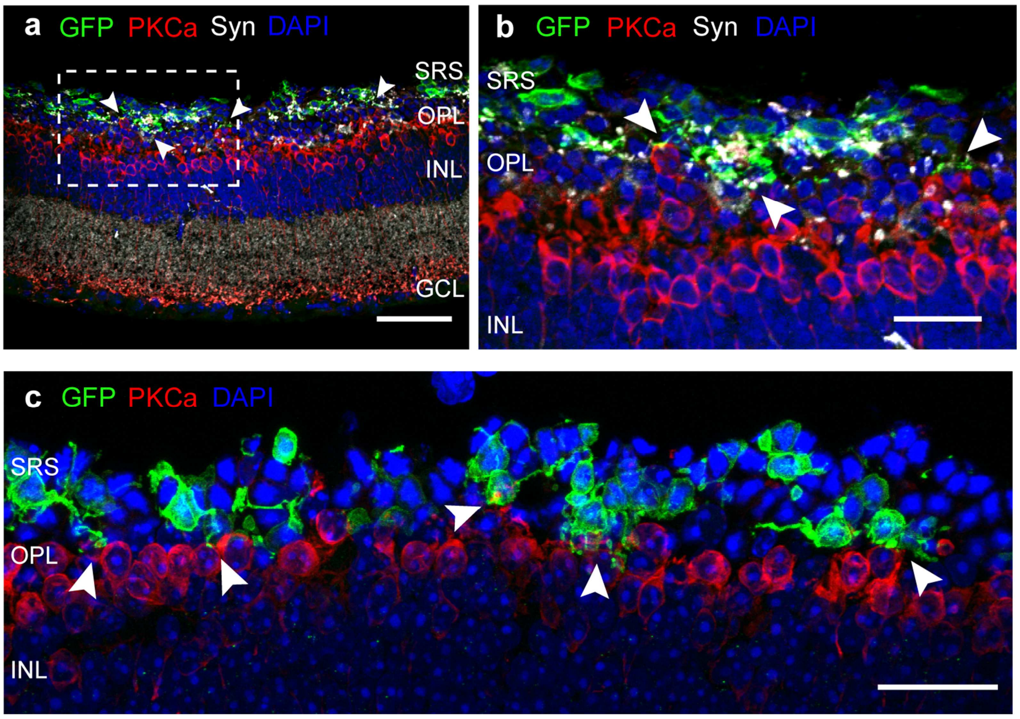

Supplementary Figure 2. Transplanted NpHR-rod precursors are located in close apposition to the host INL and express the synaptic marker synaptophysin. **a-c** *Cpfl1/Rho*$^{-/-}$ retinas transplanted with NpHR-photoreceptors showing NpHR-YFP^{+} cells (stained with anti-GFP antibody, green) located on top of host PKCα bipolar cells (red), with (**a, b**) or without synaptophysin staining (**c**), 4 weeks after transplantation. Arrows point to potential synaptic connections with host rod bipolar cells. Scale bars are 50 µm (a) and 25 µm (b, c). SRS – subretinal space, OPL – outer plexiform layer, INL – inner nuclear layer, GCL – ganglion cell layer.

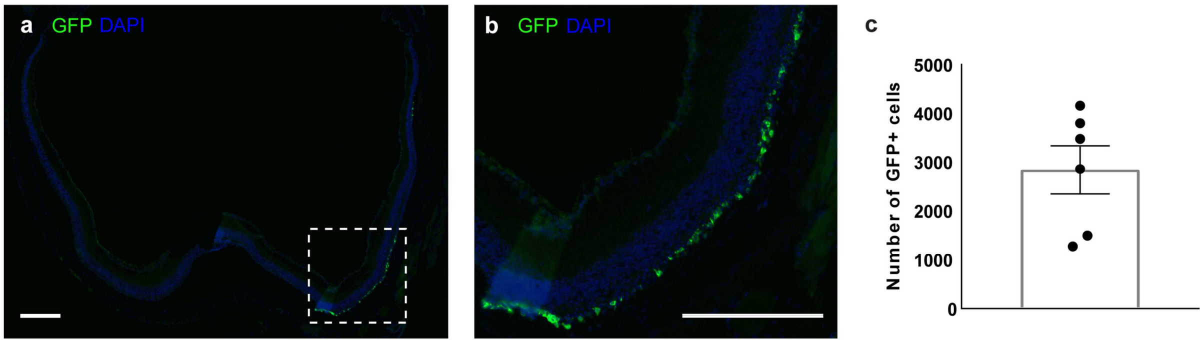

Supplementary Figure 3. Quantification of NpHR-expressing cells after transplantation of NpHR-rod precursors. **a, b** A section of a representative *Cpfl1/Rho*$^{-/-}$ retina stained with anti-GFP antibody (green) and DAPI counterstaining (blue) 4 weeks after transplantation into a P60 animal. Scale bars are 200 µm. **c** Quantification of NpHR-expressing cells from 6 individual experimental retinas (N=6). On average, 2830 ± 493 cells per retina remained in the subretinal space 4 weeks post-transplantation, corresponding to 1.42 ± 0.25% of all cells transplanted. Values are mean ± SEM with corresponding data points overlaid. Error bar is SEM. Source data are provided as a Source Data file.

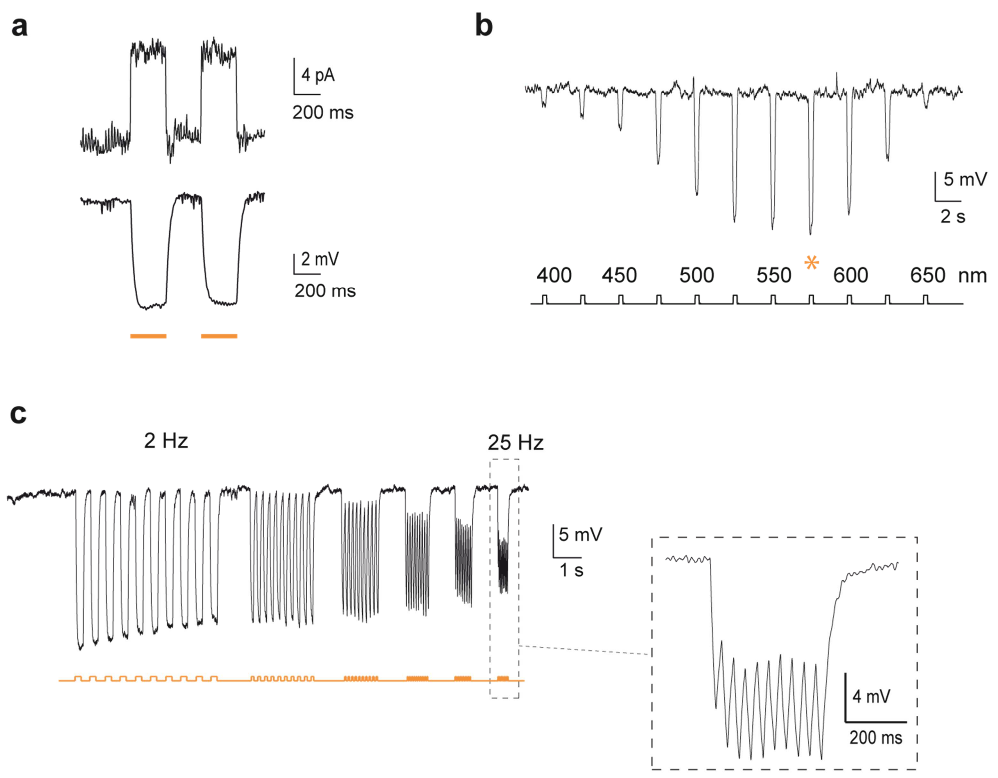

Supplementary Figure 4. NphR-expressing photoreceptor precursors transplanted into rd1 mouse respond to light. **a** Light-evoked responses of NpHR-photoreceptor stimulated with two consecutive flashes at 590 nm (top, current response; bottom, voltage response). **b** Voltage response action spectrum corresponding to a NpHR-photoreceptor stimulated at wavelengths ranging from 400 nm to 650 nm, in rd1 retina. Maximal responses were obtained at 575 nm (denoted with an orange star). **c** Temporal properties: Modulation of NpHR-induced voltage response at increasing stimulation frequencies from 2 to 25 Hz. The timing and duration of stimulation is depicted with underlying orange lines (for 590 nm stimuli; a, c) or with a black line with associated wavelengths noted above (b).

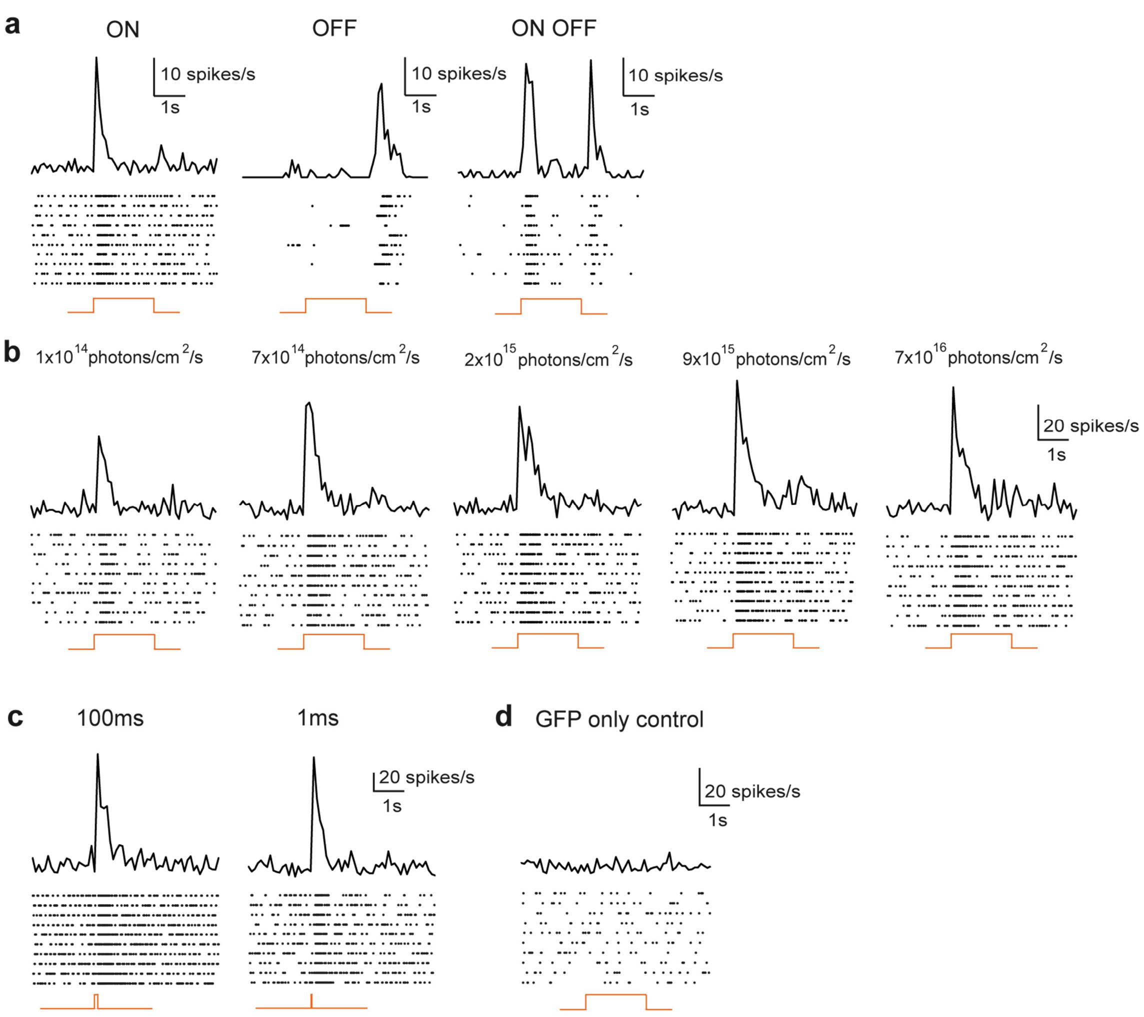

Supplementary Figure 5. Halorhodopsin-triggered RGC responses in rd1 mice. **a** RGCs firing responses shown as PSTH and raster plots recorded from transplanted rd1 mice, showing examples of cells responding with an ON-, OFF- or an ON/OFF-response (stimulation: 580 nm, 7×10^{16} photons cm^{-2} s^{-1}). **b** Responses from a representative cell at lower light intensities and **c** shorter light pulses. **d** Unresponsive cell from a control retina transplanted with GFP only photoreceptors. The timing and duration of stimulation is depicted with underlying orange lines.

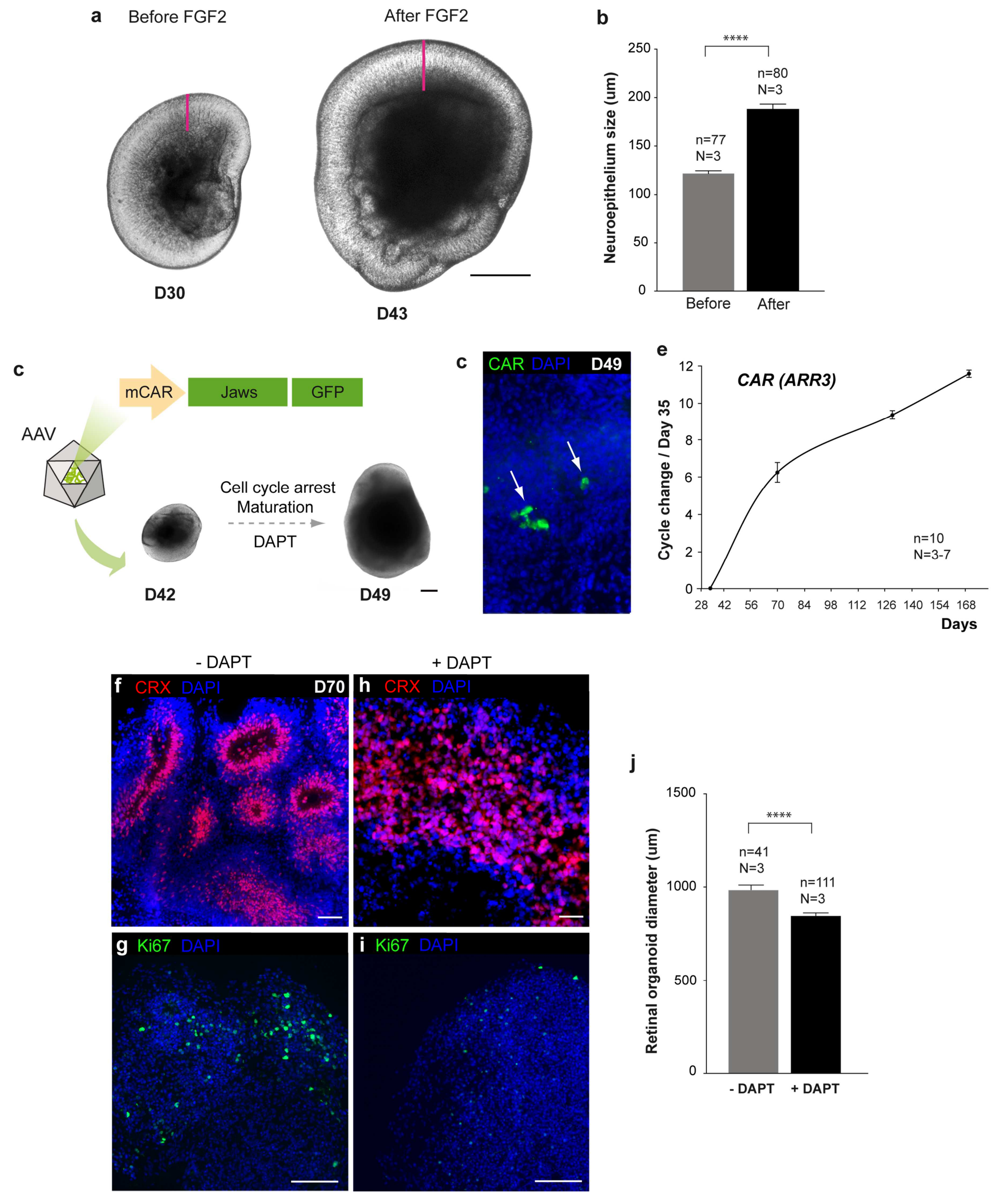

Supplementary Figure 6. Growth of the neuroepithelium in retinal organoids treated with FGF2 and the effect of Notch inhibition on retinal organoidogenesis and photoreceptor commitment. **a** Representative micrographs of a retinal organoid before (D30) and after (D43) treatment with FGF2. Pink bars show the neuroepithelium thickness quantified in b. **b** Neuroepithelium thickness before (121.7

± 2.8 μm) and after (188.7 ± 4.7 μm) addition of FGF2. **c** Schematics of the introduction of Jaws using an AAV vector and representative images of a retinal organoid before (D42) and after (D49) infection and treatment with DAPT. **d** Immunofluorescence analysis of retinal organoids showing the first CAR positive cells (green arrows) at D49 of differentiation. **e** Time course analysis of *CAR* by qPCR in differentiating retinal organoids. Data is expressed as cycle change in PCR expression level relative to D35 of differentiation. **f-i** Organoid cryosections after 70 days of differentiation without (**f, g**) or with DAPT (**h, i**). **j** Measurement of the diameter of retinal organoids on D56 of differentiation with and without DAPT treatment. Scale bars are 200 μm (a, c, f, h) and 50 μm (e, g, i). In all panels: N = number of biological replicates, n = number of organoids. Values are mean ± SEM. Error bars are SEM. Statistical significance assessed using Mann-Whitney Student's test (**** p<0,0001). Source data are provided as a Source Data file (for b, e, j).

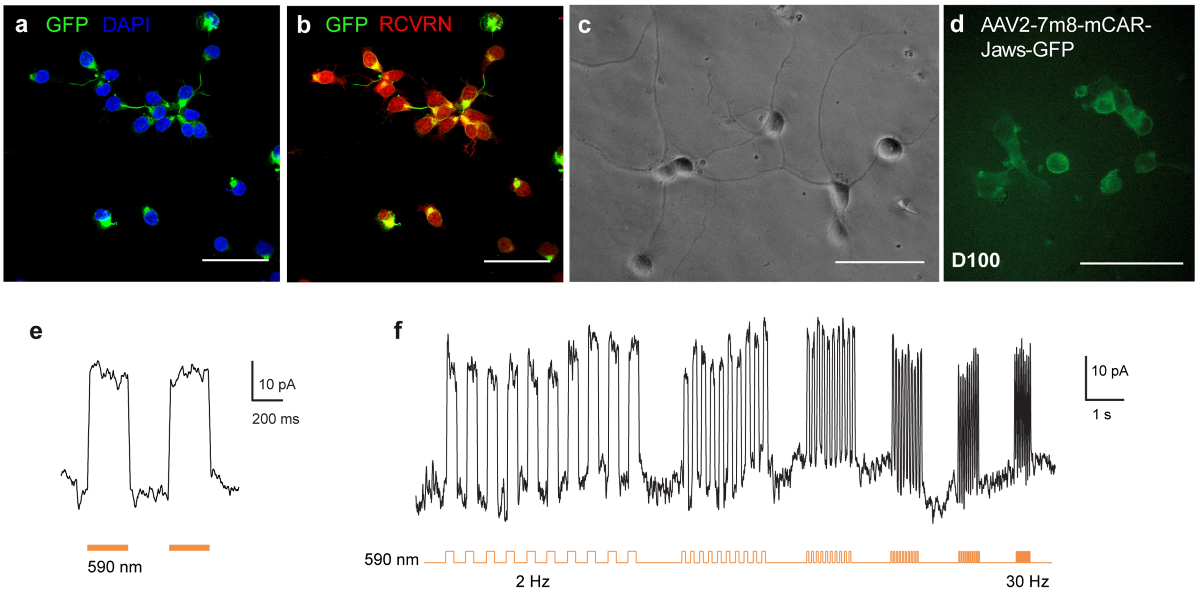

Supplementary Figure 7. Physiological analysis of monolayer cultures derived from dissociated retinal organoids. **a, b** Monolayer cultures stained with antibodies against GFP and DAPI **(a),** and photoreceptor marker RCVRN and GFP **(b)**. **c** Jaws-cones used for patch-clamp recordings in D100 monolayer cultures. **d** A two-photon laser microscope image of Jaws-cones. Scale bars are 50 µm. **e** Light-evoked photocurrent responses of Jaws-cones in the monolayer stimulated with two consecutive flashes of light at 590 nm. **f** Modulation of Jaws-induced responses at increasing stimulation frequency (2 to 30 Hz). The timing and duration of stimulation is depicted with underlying orange lines (590 nm stimulation).

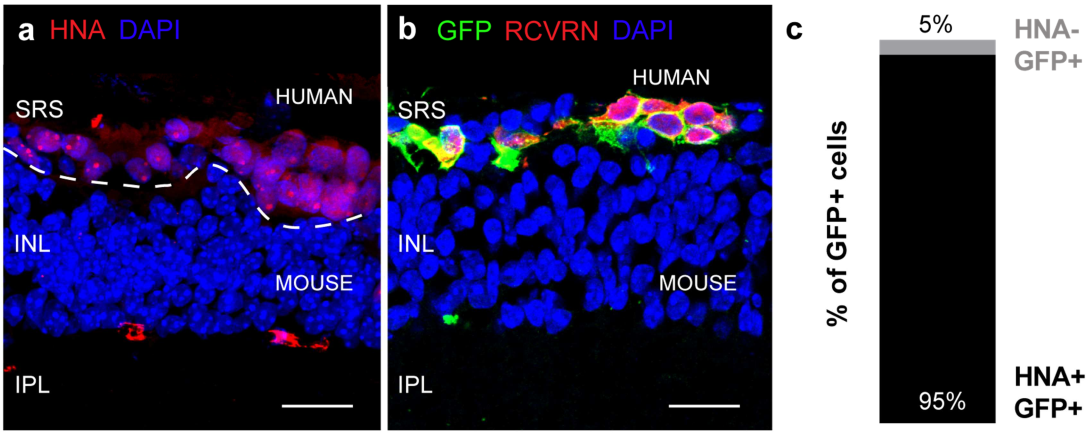

Supplementary Figure 8. Expression of human and photoreceptor markers in transplanted GFP^+ cells. **a** Immunostaining of transplanted cells against human nuclear antibody (HNA, red) showed human cells lie over the rd1 host INL. The white dashed line depicts the border between transplanted cells (top) and mouse host tissue (bottom). **b** Jaws-GFP transplanted cells co-expressed photoreceptor specific marker RCVRN (red) confirming the photoreceptor identity of GFP positive cells. Nuclei were counterstained with DAPI (blue). Scale bars are 20 μm. SRS – subretinal space, INL – inner nuclear layer, IPL – inner plexiform layer. **c** Quantification of HNA^+/GFP^+ cells, representing transplanted hiPSC-derived photoreceptors, and HNA^-/GFP^+ cells, representing cells where GFP^+ staining could be the result of material transfer, from 3 individual experimental rd1 retinas (5 weeks old at the time of transplantation; N=3). The vast majority of GFP^+ cells co-expressed HNA (95.07 ± 1.6 %; mean ± SEM). Source data are provided as a Source Data file.

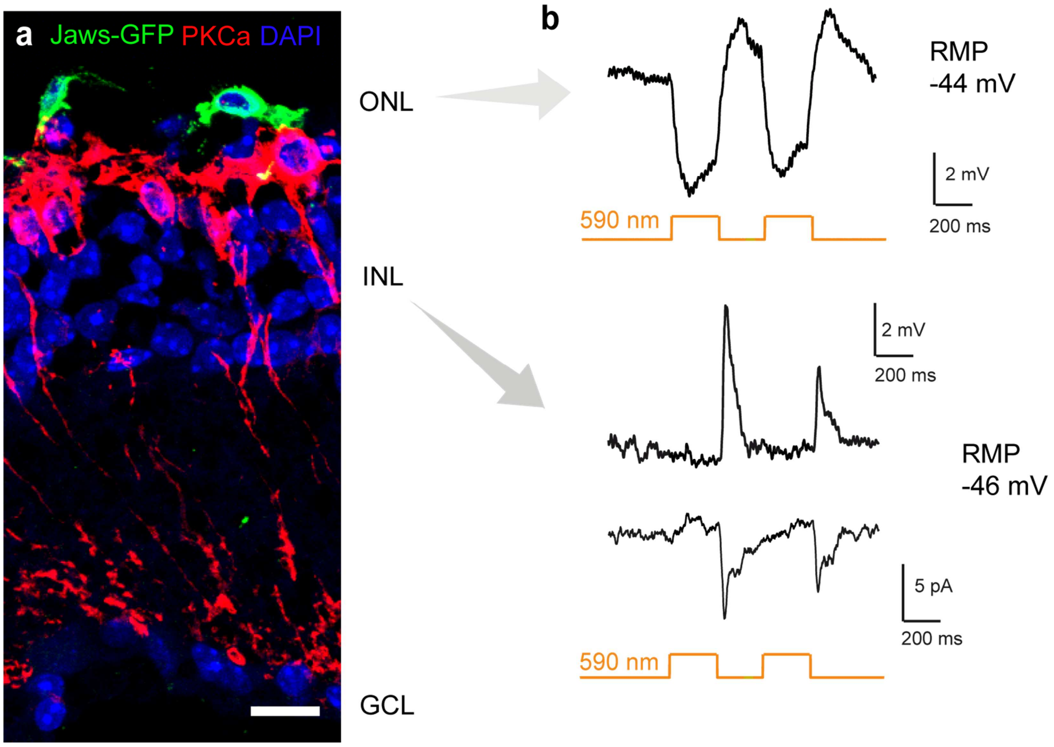

Supplementary Figure 9. Signal transduction from photoreceptors to the second order neurons in rd1 mouse transplanted with Jaws-photoreceptors. **a** An rd1 retina after transplantation, showing Jaws-cones located on top of the PKCα-stained recipient INL. ONL – outer nuclear layer, INL – inner nuclear layer, GCL – ganglion cell layer. **b** Photocurrents elicited by a Jaws-expressing donor cell transplanted in an rd1 retina (top) and the response (voltage and current) recorded from a second order OFF-neuron (bottom). The timing and duration of stimulation is depicted with underlying orange lines (590 nm stimulation).

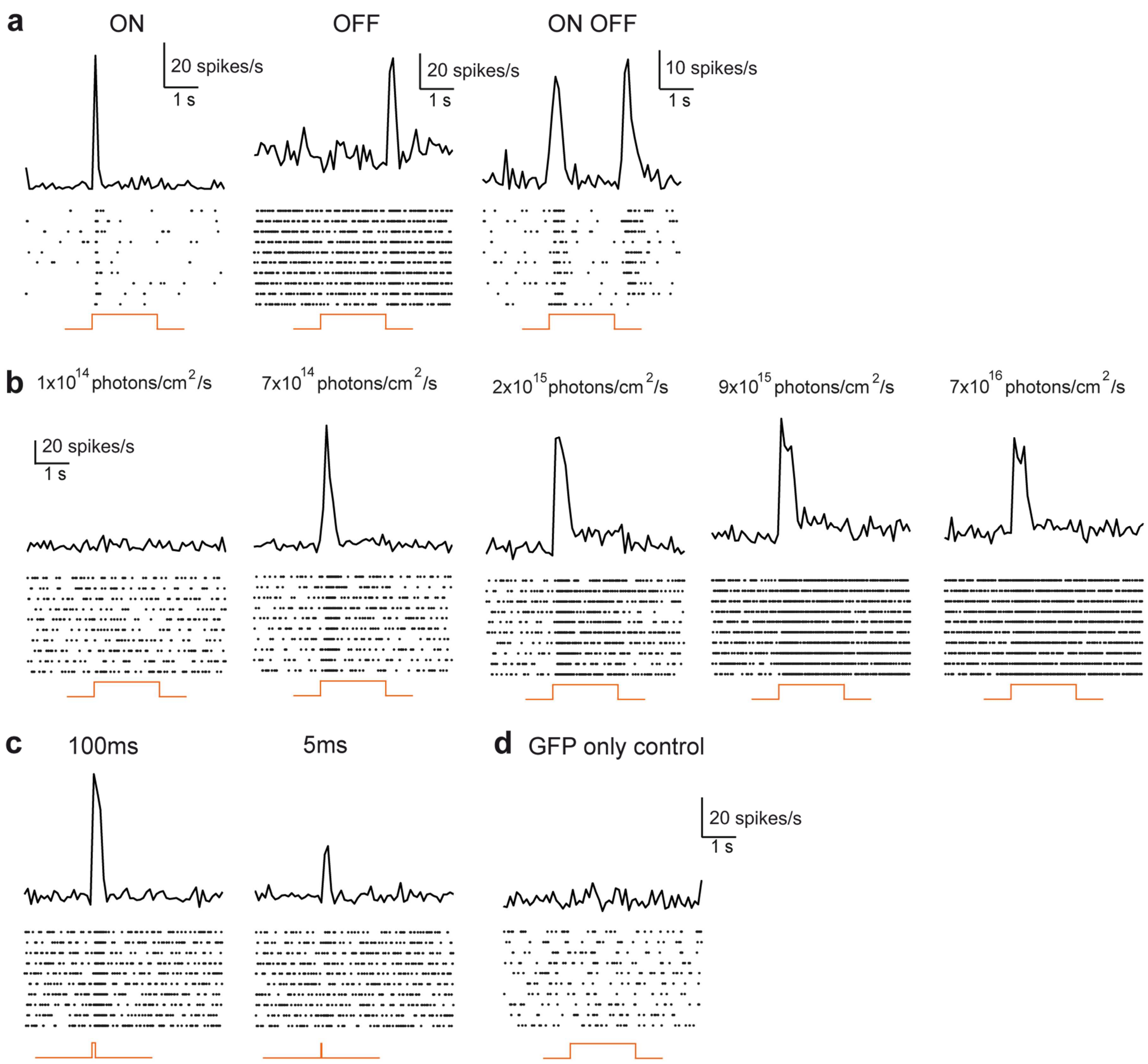

Supplementary Figure 10. Jaws-triggered RGC responses in rd1 mice. **a** RGCs firing responses shown as PSTH and raster plots recorded from transplanted rd1 mice, showing examples of cells responding with an ON-, OFF- or ON/OFF-response (stimulation: 580 nm, 7×10^{16} photons cm^{-2} s^{-1}). **b** Responses at lower light intensities and **c** shorter light pulses. **d** An unresponsive cell from a control retina transplanted with GFP only-expressing cones. The timing and duration of stimulation is depicted with underlying orange lines.

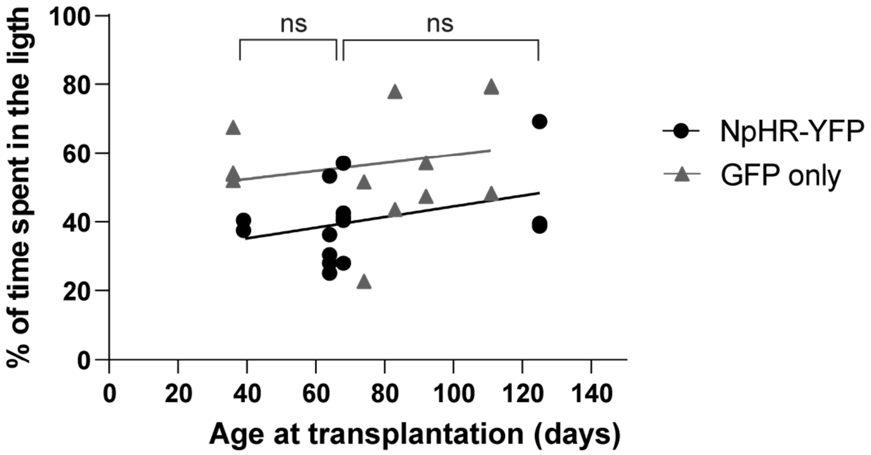

Supplementary Figure 11. The level of functional improvement is independent of the mouse host age at the time of transplantation. A diagram showing the percentage of time spent in the light compartment of the light/dark box for *Cpfl1/Rho*$^{-/-}$ mice transplanted with NpHR-YFP rods (black line with black dots) or GFP only-expressing rods (grey line with grey squares) at different ages. The difference between mice transplanted with NpHR-expressing rods at the age of 5 weeks (N=2), 9 and 10 weeks (N=10), and 18 weeks (N=3) was not significant. The trend of slightly poorer performance with older age is comparable in both the treated mice as well as controls. ns – not significant.

Supplementary Tables

Supplementary Table 1. A list of all mice used to generate figures, with specified strain, experimental group, experiment type, and ages at the time of transplantation and at the time of experiment

Fig.		*Strain*	*Experimental group*	*Experiment type*	*Age at trans-plantation (weeks)*	*Age at the time of experiment (weeks)*	*Number of mice (N)*
**Fig. 1**	**b**	*Cpfl1 Rho*$^{-/-}$	Non-injected control	IHC	-	16	1
	**c, d**	*Cpfl1 Rho*$^{-/-}$	Mouse NpHR-precursors	IHC	13	16	1
	**e**	rd1	Non-injected control	IHC	-	10	1
	**f, g**	rd1	Mouse NpHR-precursors	IHC	4	10	1
	**i**	*Cpfl1 Rho*$^{-/-}$	Mouse NpHR-precursors	FISH	9	13	3
**Fig. 2**	**a**	*Cpfl1 Rho*$^{-/-}$	Mouse NpHR-precursors	Patch-clamp	9	11	1
		Cpfl1 Rho$^{-/-}$	Mouse NpHR-precursors	Patch-clamp	10	14	1
		Cpfl1 Rho$^{-/-}$	Mouse GFP only-precursors	Patch-clamp	6.5	11	1
		rd1	Mouse NpHR-precursors	Patch-clamp	11	13	1
	**b, c**	*Cpfl1 Rho*$^{-/-}$	Mouse NpHR-precursors	Patch-clamp	10	14	1
	**d**	*Cpfl1 Rho*$^{-/-}$	Mouse NpHR-precursors	Patch-clamp	9	11	1
		Cpfl1 Rho$^{-/-}$	Mouse NpHR-precursors	Patch-clamp	10	14	1
		rd1	Mouse NpHR-precursors	Patch-clamp	11	13	1
**Fig. 3**	**a-g, i**	*Cpfl1 Rho*$^{-/-}$	Mouse NpHR-precursors	MEA	10	14	2
		Cpfl1 Rho$^{-/-}$	Mouse NpHR-precursors	MEA	10	13.5	1
		Cpfl1 Rho$^{-/-}$	Mouse NpHR-precursors	MEA	10	13	1
	**h, i**	*Cpfl1 Rho*$^{-/-}$	Mouse GFP only-precursors	MEA	5	9	2
		Cpfl1 Rho$^{-/-}$	Mouse GFP only-precursors	MEA	5.5	10	1
		Cpfl1 Rho$^{-/-}$	Mouse GFP only-precursors	MEA	6.5	11	2
		Cpfl1 Rho$^{-/-}$	Mouse GFP only-precursors	MEA	12	16	1
		Cpfl1 Rho$^{-/-}$	Mouse GFP only-	MEA	13	17	1

			precursors				
	**k**	*Cpfl1 Rho*$^{-/-}$	Non-injected control	Light/dark box	-	7.5	4
		Cpfl1 Rho$^{-/-}$	Non-injected control	Light/dark box	-	8	2
		Cpfl1 Rho$^{-/-}$	Non-injected control	Light/dark box	-	9	2
		Cpfl1 Rho$^{-/-}$	Non-injected control	Light/dark box	-	13	3
		Cpfl1 Rho$^{-/-}$	Mouse GFP only-precursors	Light/dark box	5	8	4
		Cpfl1 Rho$^{-/-}$	Mouse GFP only-precursors	Light/dark box	10.5	13.5	2
		Cpfl1 Rho$^{-/-}$	Mouse GFP only-precursors	Light/dark box	12	15	2
		Cpfl1 Rho$^{-/-}$	Mouse GFP only-precursors	Light/dark box	13	16	3
		Cpfl1 Rho$^{-/-}$	Mouse GFP only-precursors	Light/dark box	16	21	1
		Cpfl1 Rho$^{-/-}$	Mouse NpHR-precursors	Light/dark box	9	12	6
		Cpfl1 Rho$^{-/-}$	Mouse NpHR-precursors	Light/dark box	10	13.5	4
		Cpfl1 Rho$^{-/-}$	Mouse NpHR-precursors	Light/dark box	18	23	3
**Fig. 5**	**a, b**	*Cpfl1 Rho*$^{-/-}$	Human Jaws-cones	IHC	10.5	14	1
	**c**	rd1	Human Jaws-cones	IHC	5	9	1
	**d**	rd1	Human Jaws-cones	IHC	5	9	3
	**e**	*Cpfl1 Rho*$^{-/-}$	Human Jaws-cones	Patch-clamp	10.5	14	2
		Cpfl1 Rho$^{-/-}$	Human GFP only-cones	Patch-clamp	7	10	1
		rd1	Human Jaws-cones	Patch-clamp	4	8	1
		rd1	Human Jaws-cones	Patch-clamp	5	9	1
	**f, g**	*Cpfl1 Rho*$^{-/-}$	Human Jaws-cones	Patch-clamp	10.5	14.5	1
	**h**	*Cpfl1 Rho*$^{-/-}$	Human Jaws-cones	Patch-clamp	10.5	14	2
		rd1	Human Jaws-cones	Patch-clamp	4	8	1
		rd1	Human Jaws-cones	Patch-clamp	5	9	1
	**i, j**	*Cpfl1 Rho*$^{-/-}$	Human Jaws-cones	MEA	10.5	14.5	1
	**k**	*Cpfl1 Rho*$^{-/-}$	Human GFP only-cones	MEA	7	10	1

Supplementary Table 2. Media formulation

Medium	*Formulation*
**Proneural medium**	Essential 6™ Medium (Gibco, A1516401)<br>N-2 supplement (100X) 1% (Gibco, 17502048)<br>Penicillin-Streptomycin 1% (Gibco, 15140122)
**Maturation medium**	DMEM/F-12 (Gibco, 11320074)<br>B-27™ Supplement (50X), serum free 2% (Gibco, 17504044)<br>MEM Non-Essential Amino Acids Solution (100X) 1% (Gibco 11140035)<br>Penicillin-Streptomycin 1% (Gibco, 15140122)
**Ringer solution**	NaCl 155 mM, KCl 5 mM, $CaCl_2$ 2 mM, $NaCl_2$ 1 mM, NaH_2PO_4 2 mM, HEPES 10 mM, glucose 10 mM

Supplementary Table 3. List of TaqMan® Gene Expression ID Assays used for qRT-PCR

Gene symbols	*Assays IDs*
18S	*18S-Hs99999901_s1*
CONE ARRESTIN	*ARR3-Hs00182888_m1*
RECOVERIN	*RCVRN-Hs00610056_m1*

Supplementary Table 4. List of primary antibodies used for immunostaining

Antibody	*Reference*	*Catalogue number*	*Species*	*Dilution*
**hCAR**	Gift from Cheryl Craft	-	Rabbit	1:20,000
**CRX**	Abnova	H00001406-M02	Mouse	1:5,000
**GFP**	Abcam	ab13970	Chicken	1:500
**HNA**	Millipore	MAB4383	Mouse	1:200
**Ki67**	BD Pharmagen	550609	Mouse	1:200
**PKCα**	Santa Cruz	sc-208	Rabbit	1:100
**RCVRN**	Millipore	AB5585	Rabbit	1:5,000-1:2,000
**Synaptophysin**	Sigma	SAB4502906	Mouse	1:200

Supplementary References

1 Busskamp, V. *et al.* Genetic reactivation of cone photoreceptors restores visual responses in retinitis pigmentosa. *Science* **329**, 413-417, doi:10.1126/science.1190897 (2010).

2 Materials and methods.
