# Supplementary material for: Restoration of visual function by transplantation of optogenetically engineered photoreceptors
Source: Nat Commun. 2019 Oct 4;10:4524. doi: 10.1038/s41467-019-12330-2 (PMC6778196; doi:10.1038/s41467-019-12330-2)
Supplement: Supplementary file 2 — Reporting Summary [file 41467_2019_12330_MOESM2_ESM.pdf]

nature research

Corresponding author(s): Deniz Dalkara, Jens Duebel

Reporting Summary

Nature Research wishes to improve the reproducibility of the work that we publish. This form provides structure for consistency and transparency in reporting. For further information on Nature Research policies, see Authors & Referees and the Editorial Policy Checklist.

Statistical parameters

When statistical analyses are reported, confirm that the following items are present in the relevant location (e.g. figure legend, table legend, main text, or Methods section).

n/a	Confirmed	
☐	☒	The exact sample size (*n*) for each experimental group/condition, given as a discrete number and unit of measurement
☐	☒	An indication of whether measurements were taken from distinct samples or whether the same sample was measured repeatedly
☐	☒	The statistical test(s) used AND whether they are one- or two-sided<br>*Only common tests should be described solely by name; describe more complex techniques in the Methods section.*
☒	☐	A description of all covariates tested
☒	☐	A description of any assumptions or corrections, such as tests of normality and adjustment for multiple comparisons
☐	☒	A full description of the statistics including central tendency (e.g. means) or other basic estimates (e.g. regression coefficient) AND variation (e.g. standard deviation) or associated estimates of uncertainty (e.g. confidence intervals)
☐	☒	For null hypothesis testing, the test statistic (e.g. *F*, *t*, *r*) with confidence intervals, effect sizes, degrees of freedom and *P* value noted<br>*Give P values as exact values whenever suitable.*
☒	☐	For Bayesian analysis, information on the choice of priors and Markov chain Monte Carlo settings
☒	☐	For hierarchical and complex designs, identification of the appropriate level for tests and full reporting of outcomes
☒	☐	Estimates of effect sizes (e.g. Cohen's *d*, Pearson's *r*), indicating how they were calculated
☐	☒	Clearly defined error bars<br>*State explicitly what error bars represent (e.g. SD, SE, CI)*

Our web collection on statistics for biologists may be useful.

Software and code

Policy information about availability of computer code

Data collection	MEA data was collected using the MC_Rack software (MC_Rack v4.5, Multi Channel Systems).
Data analysis	MEA data was analysed using Spike2 software v.7 (Cambridge Electronic Design Ltd). The raster plots and peristimulus time histogram data were constructed in MATLAB using custom scripts from spike-sorted channels. The MATLAB code is availabe upon request. Light/dark box data was analyzed using Panlab Smart Vision Tracking Software 3.0.05 (Harvard Apparatus).

For manuscripts utilizing custom algorithms or software that are central to the research but not yet described in published literature, software must be made available to editors/reviewers upon request. We strongly encourage code deposition in a community repository (e.g. GitHub). See the Nature Research guidelines for submitting code & software for further information.

Data

Policy information about availability of data

All manuscripts must include a data availability statement. This statement should provide the following information, where applicable:

- Accession codes, unique identifiers, or web links for publicly available datasets
- A list of figures that have associated raw data
- A description of any restrictions on data availability

The data that support the findings of this study are available from the corresponding author upon reasonable request. The source data underlying Figures 1i, 2a, d, 3c, e, g, i, k, 4f, 5d, e, h, j, resting membrane potential values (RPM), and Supplementary Figures 3c, 6b, e, j and 8c are provided as a Source Data file.

Field-specific reporting

Please select the best fit for your research. If you are not sure, read the appropriate sections before making your selection.

☒ Life sciences ☐ Behavioural & social sciences ☐ Ecological, evolutionary & environmental sciences

For a reference copy of the document with all sections, see nature.com/authors/policies/ReportingSummary-flat.pdf

Life sciences study design

All studies must disclose on these points even when the disclosure is negative.

Sample size	There was no sample-size calculation performed. We used other similar studies as a guide in choosing the sample sizes.
Data exclusions	Fig. 3c. Only ON-responding RGCs were taken into account, since L-AP4 is an ON pathway blocker.<br>Fig. 3e. The graph shows data for OFF-responding cells. Because the maximal firing rates of ON-responding cells were normally higher compared to the OFF-responding cells, showing both types in the same graph would mask the significant difference resulting from the light wavelenght change.<br>Fig. 3i. The graph is comparing the firing rates of ON-responding cells from NpHR-treated mice and GFP-only treated controls. Again, for the same reason as in Fig. 2e, only one of the cell types is shown in the quantification graph.<br>Fig. 3k. The animals that never crossed the barrier in the first 3 minutes in the dark were excluded from the trial.
Replication	The reproducibility of the experimental findings was confirmed within our laboratory by doing replicates.
Randomization	Animals and cultures were equivalent and not distinguishable one from another before treatment. De facto sample randomizing was performed without the need of a formal randomization process. Later on, the samples were grouped according to the mouse model and the treatment that the mice received (for example rd1 mice treated with NpHR photoreceptor precursors, rd1 mice treated with Jaws-expressing hiPSC-derived photoreceptors, rd1 mice treated with GFP-only photoreceptors, non-injected rd1 mice, etc.).
Blinding	The investigators were not blinded during data collection or analysis. In light/dark box analysis, two people analyzed the same data and/or the data was analyzed using a software to exclude bias.

Behavioural & social sciences study design

All studies must disclose on these points even when the disclosure is negative.

Study description	*Briefly describe the study type including whether data are quantitative, qualitative, or mixed-methods (e.g. qualitative cross-sectional, quantitative experimental, mixed-methods case study).*
Research sample	*State the research sample (e.g. Harvard university undergraduates, villagers in rural India) and provide relevant demographic information (e.g. age, sex) and indicate whether the sample is representative. Provide a rationale for the study sample chosen. For studies involving existing datasets, please describe the dataset and source.*
Sampling strategy	*Describe the sampling procedure (e.g. random, snowball, stratified, convenience). Describe the statistical methods that were used to predetermine sample size OR if no sample-size calculation was performed, describe how sample sizes were chosen and provide a rationale for why these sample sizes are sufficient. For qualitative data, please indicate whether data saturation was considered, and what criteria were used to decide that no further sampling was needed.*
Data collection	*Provide details about the data collection procedure, including the instruments or devices used to record the data (e.g. pen and paper, computer, eye tracker, video or audio equipment) whether anyone was present besides the participant(s) and the researcher, and whether the researcher was blind to experimental condition and/or the study hypothesis during data collection.*
Timing	*Indicate the start and stop dates of data collection. If there is a gap between collection periods, state the dates for each sample cohort.*

Data exclusions	*If no data were excluded from the analyses, state so OR if data were excluded, provide the exact number of exclusions and the rationale behind them, indicating whether exclusion criteria were pre-established.*
Non-participation	*State how many participants dropped out/declined participation and the reason(s) given OR provide response rate OR state that no participants dropped out/declined participation.*
Randomization	*If participants were not allocated into experimental groups, state so OR describe how participants were allocated to groups, and if allocation was not random, describe how covariates were controlled.*

Ecological, evolutionary & environmental sciences study design

All studies must disclose on these points even when the disclosure is negative.

Study description	*Briefly describe the study. For quantitative data include treatment factors and interactions, design structure (e.g. factorial, nested, hierarchical), nature and number of experimental units and replicates.*
Research sample	*Describe the research sample (e.g. a group of tagged Passer domesticus, all Stenocereus thurberi within Organ Pipe Cactus National Monument), and provide a rationale for the sample choice. When relevant, describe the organism taxa, source, sex, age range and any manipulations. State what population the sample is meant to represent when applicable. For studies involving existing datasets, describe the data and its source.*
Sampling strategy	*Note the sampling procedure. Describe the statistical methods that were used to predetermine sample size OR if no sample-size calculation was performed, describe how sample sizes were chosen and provide a rationale for why these sample sizes are sufficient.*
Data collection	*Describe the data collection procedure, including who recorded the data and how.*
Timing and spatial scale	*Indicate the start and stop dates of data collection, noting the frequency and periodicity of sampling and providing a rationale for these choices. If there is a gap between collection periods, state the dates for each sample cohort. Specify the spatial scale from which the data are taken*
Data exclusions	*If no data were excluded from the analyses, state so OR if data were excluded, describe the exclusions and the rationale behind them, indicating whether exclusion criteria were pre-established.*
Reproducibility	*Describe the measures taken to verify the reproducibility of experimental findings. For each experiment, note whether any attempts to repeat the experiment failed OR state that all attempts to repeat the experiment were successful.*
Randomization	*Describe how samples/organisms/participants were allocated into groups. If allocation was not random, describe how covariates were controlled. If this is not relevant to your study, explain why.*
Blinding	*Describe the extent of blinding used during data acquisition and analysis. If blinding was not possible, describe why OR explain why blinding was not relevant to your study.*

Did the study involve field work? ☐ Yes ☐ No

Field work, collection and transport

Field conditions	*Describe the study conditions for field work, providing relevant parameters (e.g. temperature, rainfall).*
Location	*State the location of the sampling or experiment, providing relevant parameters (e.g. latitude and longitude, elevation, water depth).*
Access and import/export	*Describe the efforts you have made to access habitats and to collect and import/export your samples in a responsible manner and in compliance with local, national and international laws, noting any permits that were obtained (give the name of the issuing authority, the date of issue, and any identifying information).*
Disturbance	*Describe any disturbance caused by the study and how it was minimized.*

Reporting for specific materials, systems and methods

Materials & experimental systems

n/a	Involved in the study	
☐	☒	Unique biological materials
☐	☒	Antibodies
☐	☒	Eukaryotic cell lines
☒	☐	Palaeontology
☐	☒	Animals and other organisms
☒	☐	Human research participants

Methods

n/a	Involved in the study	
☒	☐	ChIP-seq
☒	☐	Flow cytometry
☒	☐	MRI-based neuroimaging

Unique biological materials

Policy information about availability of materials

Obtaining unique materials	All unique materials are readily availabe from the authors or from standard commerial sources. The commercial sources are specified in the Methods section and Supplementary Tables 2 and 4.

Antibodies

Antibodies used

All the antibodies used are listed in Supplementary Table 4.

Validation

hCAR
provided by Cheryl Craft, University of Southern California

CRX
http://www.abnova.com/products/products_detail.asp?catalog_id=H00001406-M02
Application: WB
Supplied name: CRX monoclonal antibody (M02)
Catalogue number: H00001406-M02
Clone name: 4G11
Lot number: GA211-4G11
Species reactivity: human

GFP
https://www.abcam.com/gfp-antibody-ab13970.html
Application: IHC-P, WB, IHC - wholemount, IHC-FrFl, ICC/IF, IHC-Fr, IHC-FoFr
Supplied name: Anti-GFP antibody
Catalogue number: ab13970
Clone name: not provided
Lot number: several
Species reactivity: NA

HNA
http://www.merckmillipore.com/FR/fr/product/Anti-Nuclei-Antibody-clone-3E1.3,MM_NF-MAB4383
Applications: FC, IC, IH
Supplied name: Anti-Nuclei Antibody
Catalogue number: MAB4383
Clone name: 3EI.3
Lot number: 2792243
Species reactivity: human

Ki67
http://www.bdbiosciences.com/us/applications/research/intracellular-flow/intracellular-antibodies-and-isotype-controls/anti-rat-antibodies/purified-mouse-anti-ki-67-b56/p/550609
Applications: Flow cytometry, Immunohistochemistry (Tested During Development)
Supplied name: MKI67
Catalogue number: 550609
Clone name: Clone B56
Lot number: 6064856
Species reactivity: human (QC testing), mouse (tested in development), rat, rhesus

PKC alpha
No longer produced
https://www.scbt.com/scbt/fr/product/pkc-alpha-antibody-c-20
Applications: WB, IP, IF, ELISA
Supplied name: PKC alpha
Catalogue number: sc-208
Clone name: C-20
Lot number: H1512
Species reactivity: mouse, rat, human

RCVRN
http://www.merckmillipore.com/FR/fr/product/Anti-Recoverin-Antibody, MM_NF-AB5585
Applications: ICC, IHC, IH(P), WB
Supplied name: Anti-Recoverin Antibody
Catalogue number: AB5585
Clone name: not provided
Lot number: 2776840
Species reactivity: human, mouse, rat, chicken

Synaptophysin https://www.sigmaaldrich.com/catalog/product/sigma/sab4502906?
lang=fr&region=FR&gclid=EAIaIQobChMI6v6as4TP3gIVl4XVCh3glQlLEAAYASAAEgLbqPD_BwE
Applications: ELISA, immunohistochemistry, WB

Supplied name: Anti-Synaptophysin antibody produced in rabbit
Catalogue number: SAB4502906
Clone name: SVP-38
Lot number: 026M4788V
Species reactivity: rat, mouse, human

Eukaryotic cell lines

Policy information about cell lines

Cell line source(s)	All experiments were carried out using hiPSC-2 cell line, previously established from human fibroblasts (Reichman et al., 2014).
Authentication	Cell line is not authenticated but the characterization of the hiPSC-2 cell line consisted of positive alkaline phosphatase staining, immunohistochemistry, qRT-PCR analysis of pluripotency markers (NANOG, TRA1-81, OCT4, and SSEA4), capacity for embryoid body formation, differentiation towards the three main germ layers markers (endoderm, SOX17, mesoderm, BRACHYURY, SMA, and ectoderm PAX6, TUJ1), teratome formation in NSG mouse and karyotype analysis (Reichman et al., 2014).
Mycoplasma contamination	Absence of mycoplasma contamination was verified by theMycoAlert™Mycoplasma Detection Kit (selective biochemical test of mycoplasma enzymes) used according to the manufacturer's instructions (Lonza).
Commonly misidentified lines (See ICLAC register)	No commonly misidentified cell lines used.

Palaeontology

Specimen provenance	*Provide provenance information for specimens and describe permits that were obtained for the work (including the name of the issuing authority, the date of issue, and any identifying information).*
Specimen deposition	*Indicate where the specimens have been deposited to permit free access by other researchers.*
Dating methods	*If new dates are provided, describe how they were obtained (e.g. collection, storage, sample pretreatment and measurement), where they were obtained (i.e. lab name), the calibration program and the protocol for quality assurance OR state that no new dates are provided.*

☐ Tick this box to confirm that the raw and calibrated dates are available in the paper or in Supplementary Information.

Animals and other organisms

Policy information about studies involving animals; ARRIVE guidelines recommended for reporting animal research

Laboratory animals	Wild type C57BL/6 mice of both sexes age 4 days were used as a source of photoreceptor precursor donor cells. Two mouse models were used as photoreceptor tranplant recipients: Cone photoreceptor function loss 1/rhodopsin-deficient double-mutant (Cpfl1/Rho−/−) mouse and retinal degeneration 1 (rd1) mouse (C3Hrd/rd). Mice of both sexes were used in all of the experiments. At the time of transplantation, the Cpfl1/Rho−/− mice were 9-18 weeks of age and the rd1 mice were 4-11 weeks of age. The experiments were performed at least 3 weeks after transplantation. Laboratory mice were housed under a 12-hour light-dark cycle with free access to food and water.
Wild animals	The study did not involve wild animals.
Field-collected samples	The study did not involve field-collected samples.

Human research participants

Policy information about studies involving human research participants

Population characteristics	*Describe the covariate-relevant population characteristics of the human research participants (e.g. age, gender, genotypic information, past and current diagnosis and treatment categories). If you filled out the behavioural & social sciences study design questions and have nothing to add here, write "See above."*
Recruitment	*Describe how participants were recruited. Outline any potential self-selection bias or other biases that may be present and how these are likely to impact results.*

ChIP-seq

Data deposition

☐ Confirm that both raw and final processed data have been deposited in a public database such as GEO.

☐ Confirm that you have deposited or provided access to graph files (e.g. BED files) for the called peaks.

Data access links<br>May remain private before publication.	*For "Initial submission" or "Revised version" documents, provide reviewer access links. For your "Final submission" document, provide a link to the deposited data.*
Files in database submission	*Provide a list of all files available in the database submission.*
Genome browser session (e.g. UCSC)	*Provide a link to an anonymized genome browser session for "Initial submission" and "Revised version" documents only, to enable peer review. Write "no longer applicable" for "Final submission" documents.*

Methodology

Replicates	*Describe the experimental replicates, specifying number, type and replicate agreement.*
Sequencing depth	*Describe the sequencing depth for each experiment, providing the total number of reads, uniquely mapped reads, length of reads and whether they were paired- or single-end.*
Antibodies	*Describe the antibodies used for the ChIP-seq experiments; as applicable, provide supplier name, catalog number, clone name, and lot number.*
Peak calling parameters	*Specify the command line program and parameters used for read mapping and peak calling, including the ChIP, control and index files used.*
Data quality	*Describe the methods used to ensure data quality in full detail, including how many peaks are at FDR 5% and above 5-fold enrichment.*
Software	*Describe the software used to collect and analyze the ChIP-seq data. For custom code that has been deposited into a community repository, provide accession details.*

Flow Cytometry

Plots

Confirm that:

☐ The axis labels state the marker and fluorochrome used (e.g. CD4-FITC).

☐ The axis scales are clearly visible. Include numbers along axes only for bottom left plot of group (a 'group' is an analysis of identical markers).

☐ All plots are contour plots with outliers or pseudocolor plots.

☐ A numerical value for number of cells or percentage (with statistics) is provided.

Methodology

Sample preparation	*Describe the sample preparation, detailing the biological source of the cells and any tissue processing steps used.*
Instrument	*Identify the instrument used for data collection, specifying make and model number.*
Software	*Describe the software used to collect and analyze the flow cytometry data. For custom code that has been deposited into a community repository, provide accession details.*
Cell population abundance	*Describe the abundance of the relevant cell populations within post-sort fractions, providing details on the purity of the samples and how it was determined.*

Gating strategy	*Describe the gating strategy used for all relevant experiments, specifying the preliminary FSC/SSC gates of the starting cell population, indicating where boundaries between "positive" and "negative" staining cell populations are defined.*

☐ Tick this box to confirm that a figure exemplifying the gating strategy is provided in the Supplementary Information.

Magnetic resonance imaging

Experimental design

Design type	*Indicate task or resting state; event-related or block design.*
Design specifications	*Specify the number of blocks, trials or experimental units per session and/or subject, and specify the length of each trial or block (if trials are blocked) and interval between trials.*
Behavioral performance measures	*State number and/or type of variables recorded (e.g. correct button press, response time) and what statistics were used to establish that the subjects were performing the task as expected (e.g. mean, range, and/or standard deviation across subjects).*

Acquisition

Imaging type(s)	*Specify: functional, structural, diffusion, perfusion.*
Field strength	*Specify in Tesla*
Sequence & imaging parameters	*Specify the pulse sequence type (gradient echo, spin echo, etc.), imaging type (EPI, spiral, etc.), field of view, matrix size, slice thickness, orientation and TE/TR/flip angle.*
Area of acquisition	*State whether a whole brain scan was used OR define the area of acquisition, describing how the region was determined.*

Diffusion MRI ☐ Used ☐ Not used

Preprocessing

Preprocessing software	*Provide detail on software version and revision number and on specific parameters (model/functions, brain extraction, segmentation, smoothing kernel size, etc.).*
Normalization	*If data were normalized/standardized, describe the approach(es): specify linear or non-linear and define image types used for transformation OR indicate that data were not normalized and explain rationale for lack of normalization.*
Normalization template	*Describe the template used for normalization/transformation, specifying subject space or group standardized space (e.g. original Talairach, MNI305, ICBM152) OR indicate that the data were not normalized.*
Noise and artifact removal	*Describe your procedure(s) for artifact and structured noise removal, specifying motion parameters, tissue signals and physiological signals (heart rate, respiration).*
Volume censoring	*Define your software and/or method and criteria for volume censoring, and state the extent of such censoring.*

Statistical modeling & inference

Model type and settings	*Specify type (mass univariate, multivariate, RSA, predictive, etc.) and describe essential details of the model at the first and second levels (e.g. fixed, random or mixed effects; drift or auto-correlation).*
Effect(s) tested	*Define precise effect in terms of the task or stimulus conditions instead of psychological concepts and indicate whether ANOVA or factorial designs were used.*

Specify type of analysis: ☐ Whole brain ☐ ROI-based ☐ Both

Statistic type for inference (See Eklund et al. 2016)	*Specify voxel-wise or cluster-wise and report all relevant parameters for cluster-wise methods.*
Correction	*Describe the type of correction and how it is obtained for multiple comparisons (e.g. FWE, FDR, permutation or Monte Carlo).*

Models & analysis

n/a	Involved in the study
☐	☐ Functional and/or effective connectivity
☐	☐ Graph analysis
☐	☐ Multivariate modeling or predictive analysis

Functional and/or effective connectivity	*Report the measures of dependence used and the model details (e.g. Pearson correlation, partial correlation, mutual information).*
Graph analysis	*Report the dependent variable and connectivity measure, specifying weighted graph or binarized graph, subject- or group-level, and the global and/or node summaries used (e.g. clustering coefficient, efficiency, etc.).*
Multivariate modeling and predictive analysis	*Specify independent variables, features extraction and dimension reduction, model, training and evaluation metrics.*
